# Supplementary material for: Monoclonal Antibodies against Calcitonin Gene-Related Peptide for Migraine Prophylaxis: A Systematic Review of Real-World Data
Source: Cells. 2022 Dec 29;12(1):143. doi: 10.3390/cells12010143 (PMC9819019; doi:10.3390/cells12010143)
Supplement: Supplementary file 1 [file cells-12-00143-s001.zip › cells-1982935-supplementary.pdf]

Supplementary Table S1. Characteristics reported in clinic-based studies.

Ref.	Patients			Migraine diagnosis		Headache				Anti-CGRP-mAb			Established prophylactics	
	Patients	Women	Age (years)	Any	ICD	EM	CM	Aura	MOH	E	F	G	Current use	Prior failure
	n	%	mean	+/-	+/-	+/-	+/-	+/-	+/-	+/-	+/-	+/-	+/-	+/-
[39]	77	84.42	40	+	+	-	+	+	+	+	+	+	+	-
[40]	78	85.90	49	+	+	-	+	N/A	N/A	+	-	-	+	+
[41]	111	79.28	51	+	+	-	+	+	+	+	-	-	+	-
[42]	210	86.67	46	+	+	+	+	+	+	+	-	-	+	+
[43]	172	82.56	50	+	+	-	+	N/A	N/A	+	+	+	+	+
[44]	81	80.25	50	+	+	-	+	+	+	+	-	-	+	+
[45]	66	93.94	46	+	+	-	+	N/A	N/A	+	+	+	+	+
[46]	145	89.66	40	+	N/A	+	+	+	+	+	-	-	+	-
[47]	91	85.71	39	+	+	+	+	+	+	+	-	-	+	-
[48]	1003	75.77	40	+	+	+	+	N/A	N/A	-	+	-	+	+
[49]	18	83.33	49	+	+	+	+	+	+	+	-	-	+	-
[50]	1034	86.36	48	+	+	-	+	+	+	+	-	-	+	+
[51]	52	90.38	48	+	+	+	+	+	+	+	-	+	+	-
[52]	418	62.20	49	+	+	N/A	N/A	N/A	N/A	+	-	-	N/A	-
[53]	30	80.00	N/A	+	+	+	+	+	+	-	-	+	+	-
[54]	26	96.15	51	+	+	+	+	+	+	+	-	-	+	+
[55]	148	93.24	47	+	+	-	+	N/A	N/A	+	+	+	+	+
[56]	50	N/A	N/A	+	+	-	+	N/A	N/A	+	-	-	N/A	+
[57]	1410	81.84	48	+	+	+	+	+	+	+	-	-	+	-
[58]	1215	81.07	N/A	+	+	+	+	N/A	N/A	+	-	-	+	-
[59]	18	100.00	37	+	+	-	+	+	+	+	-	-	+	+
[60]	89	87.64	47	+	+	+	+	+	+	+	-	-	+	+
[61]	25	68.00	47	+	+	+	+	+	+	+	+	+	+	-

[62]	139	83.45	53	+	+	-	+	N/A	N/A	+	-	-	+	+
[63]	101	89.11	49	+	+	+	+	+	+	+	-	-	+	-
[64]	30	80.00	50	+	+	+	+	N/A	N/A	+	-	+	+	-
[65]	32	75.00	43	+	+	N/A	N/A	+	+	+	+	+	+	+
[66]	100	82.00	48	+	+	+	+	+	+	+	-	-	+	+
[67]	90	91.11	N/A	+	+	+	+	+	+	+	+	-	+	+
[68]	82	82.93	51	+	+	+	+	+	+	+	-	-	+	-
[69]	17	94.12	n/a	+	+	-	+	N/A	N/A	+	+	+	+	+
[70]	142	N/A	46	+	+	+	+	N/A	N/A	+	-	+	+	+
[71]	78	N/A	47	+	+	+	+	N/A	N/A	+	-	-	+	-
[72]	372	76.34	48	+	+	+	+	N/A	N/A	+	-	-	+	+
[73]	242	71.07	48	+	+	+	+	+	+	+	-	-	+	+
[74]	53	77.36	48	+	+	+	+	+	+	-	+	-	+	+
[75]	95	80.00	41	+	+	+	+	+	+	+	-	-	+	-
[76]	139	81.29	47	+	+	-	+	+	+	+	-	+	+	+
[77]	170	90.00	47	+	+	-	+	+	+	+	-	-	+	+
[78]	42	78.57	N/A	+	+	+	+	+	+	+	+	+	+	-
[79]	32	84.38	N/A	+	+	+	+	+	+	+	-	-	N/A	N/A
[80]	100	85.00	43	+	+	+	+	-	-	+	-	-	+	-
[81]	91	86.81	47	+	+	+	+	+	+	+	-	-	+	-
[82]	185	77.30	51	+	+	-	+	+	+	+	-	-	+	N/A
[83]	44	79.55	47	+	+	-	+	+	+	+	-	+	+	+
[84]	87	83.91	42	+	+	+	+	+	+	-	-	+	+	-
[85]	162	83.33	46	+	+	-	+	+	+	+	-	-	+	-
[86]	54	94.44	45	+	+	-	+	N/A	N/A	+	-	-	+	-
[87]	159	75.47	50	+	+	+	+	+	+	+	-	-	+	-
[88]	91	87.91	N/A	+	+	-	+	+	+	+	-	-	+	+
[89]	149	77.85	52	+	+	-	+	+	+	+	-	-	+	-
[90]	39	64.10	50	+	+	-	+	+	+	+	-	-	+	+

[91]	39	94.87	51	+	+	+	+	N/A	N/A	+	+	+	+	-
[92]	70	78.57	47	+	+	-	+	+	+	+	-	-	+	+
[93]	15	66.67	51	+	+	-	+	N/A	N/A	+	-	-	N/A	-
[94]	156	82.05	44	+	+	+	+	+	+	+	-	-	+	-
[95]	98	82.65	50	+	+	-	+	+	+	+	-	-	N/A	-
[96]	155	81.29	46	+	+	+	+	+	+	+	-	+	+	+
[97]	16	87.50	44	+	+	-	+	+	+	+	-	-	+	+
[98]	163	80.37	47	+	+	+	+	+	+	-	-	+	+	-
[99]	156	82.69	47	+	+	-	+	+	+	-	-	+	+	-
[100]	154	72.08	48	+	+	+	+	+	+	+	-	+	+	-
[101]	110	82.73	47	+	+	+	+	+	+	+	-	-	+	+

Abbreviations: E - Erenumab, F - Fremanezumab, G - Galcanezumab, ICHD-3 - International Classification of Headache Disorders, 3rd edition. + reported, - not reported, N/A - information not available, EM - episodic migraine, CM - chronic migraine, MOH - medication overuse headache, ICD - International Statistical Classification of Diseases and Related Health Problems.

Supplementary Table S2. Outcome variables reported in clinic-based studies.

	Baseline			Follow-up							
Ref.	MMD	MHD	MDwAM	MMD	MHD	MDwAM	50% response	Pause	Switch	Disconti-nuation	Adverse events
[39]	-	+	-	-	+	-	+	N/A	+	+	+
[40]	+	+	-	-	-	-	+	N/A	-	-	N/A
[41]	-	+	+	-	-	-	+	+	-	+	+
[42]	+	+	-	+	+	-	+	N/A	-	+	+
[43]	-	+	-	-	-	-	+	N/A	+	+	+
[44]	-	+	+	-	+	+	+	+	-	+	+
[45]	-	+	-	-	+	-	-	N/A	-	+	+
[46]	+	+	-	-	-	-	+	+	+	+	+
[47]	-	-	-	-	-	-	+	N/A	-	-	+
[48]	+	+	-	-	-	-	+	N/A	-	+	-
[49]	+	-	+	-	-	-	+	N/A	-	+	+
[50]	+	-	-	+	-	-	+	N/A	-	+	+
[51]	+	-	-	+	-	-	+	+	-	+	+
[52]	-	-	-	-	-	-	+	N/A	+	+	+
[53]	-	-	-	-	-	-	-	N/A	-	+	+
[54]	+	-	+	-	-	-	+	N/A	-	-	-
[55]	-	+	-	-	-	-	+	N/A	+	+	+
[56]	-	-	-	-	-	-	-	N/A	-	-	+
[57]	+	+	+	-	-	-	+	+	-	+	+
[58]	-	-	-	-	-	-	+	N/A	-	+	-
[59]	-	-	-	-	-	-	+	N/A	-	-	-
[60]	+	-	-	-	-	-	+	N/A	-	+	+
[61]	-	+	-	-	-	-	+	+	+	+	-
[62]	-	-	-	-	-	-	+	N/A	-	+	+
[63]	-	-	-	-	-	-	+	N/A	+	+	+

[64]	+	-	+	+	-	+	+	N/A	+	+	+
[65]	-	-	-	-	-	-	+	N/A	+	+	+
[66]	-	-	-	-	-	-	+	+	-	+	+
[67]	-	-	-	-	-	-	+	+	-	+	+
[68]	-	+	-	-	-	-	+	N/A	-	-	-
[69]	-	+	-	-	-	-	-	N/A	-	-	+
[70]	-	-	-	-	-	-	+	N/A	-	+	+
[71]	-	-	-	-	-	-	+	N/A	-	+	+
[72]	-	-	-	-	-	-	+	+	-	+	+
[73]	-	-	-	-	-	-	+	+	-	+	+
[74]	+	-	-	-	-	-	+	N/A	-	-	+
[75]	+	+	+	+	+	+	+	N/A	-	+	+
[76]	-	-	-	-	-	-	+	N/A	-	+	+
[77]	+	+	-	+	+	-	+	N/A	-	+	+
[78]	-	-	-	-	-	-	+	N/A	-	-	+
[79]	-	-	-	-	-	-	+	+	-	+	N/A
[80]	+	-	+	+	-	+	+	N/A	-	+	+
[81]	-	-	-	-	-	-	+	N/A	-	-	N/A
[82]	-	-	-	-	-	-	-	+	-	+	N/A
[83]	+	-	+	-	-	-	+	+	-	-	+
[84]	-	+	+	-	+	+	+	N/A	-	+	+
[85]	+	+	+	+	+	+	+	N/A	-	+	+
[86]	+	+	-	-	-	-	+	N/A	-	+	+
[87]	+	-	-	-	-	-	+	N/A	-	+	+
[88]	-	-	-	-	-	-	+	N/A	-	+	+
[89]	-	+	-	-	+	-	+	N/A	-	+	+
[90]	-	-	-	-	-	-	+	N/A	-	-	+
[91]	-	-	-	-	-	-	+	+	+	-	-
[92]	-	+	-	-	+	-	+	N/A	-	+	+

[93]	+	-	-	+	-	-	+	N/A	-	-	+
[94]	-	-	-	-	-	-	+	+	-	+	+
[95]	-	-	-	-	-	-	-	+	-	+	+
[96]	+	+	+	+	+	+	+	N/A	-	-	+
[97]	-	-	+	-	-	+	-	N/A	-	-	+
[98]	-	-	-	-	-	-	+	N/A	-	+	-
[99]	-	-	-	-	-	-	+	N/A	-	-	+
[100]	-	-	-	-	-	-	+	+	-	+	-
[101]	+	-	-	+	-	-	+	N/A	-	-	+

Abbreviations: + reported, - not reported, N/A not available, MMD monthly migraine days, MHD monthly headache days, MDwAM monthly days with acute medication.
